# Antimicrobial Proteins and Peptides in Early Life: Ontogeny and Translational Opportunities

**DOI:** 10.3389/fimmu.2016.00309

**Published:** 2016-08-18

**Authors:** Anna J. Battersby, Jasmeet Khara, Victoria J. Wright, Ofer Levy, Beate Kampmann

**Affiliations:** ^1^Academic Paediatrics, Imperial College London, London, UK; ^2^Medical Research Council (MRC) Unit, Vaccines and Immunity Theme, Fajara, Gambia; ^3^Department of Pharmacy, National University of Singapore, Singapore; ^4^Precision Vaccines Program, Department of Medicine, Division of Infectious Diseases, Boston Children’s Hospital, Boston, MA, USA; ^5^Harvard Medical School, Boston, MA, USA

**Keywords:** antimicrobial peptides, protein, peptide, infant, newborn, infection, innate, immunity

## Abstract

While developing adaptive immune responses, young infants are especially vulnerable to serious infections, including sepsis, meningitis, and pneumonia. Antimicrobial proteins and peptides (APPs) are key effectors that function as broad-spectrum anti-infectives. This review seeks to summarize the clinically relevant functional qualities of APPs and the increasing clinical trial evidence for their use to combat serious infections in infancy. Levels of APPs are relatively low in early life, especially in infants born preterm or with low birth weight (LBW). There are several rationales for the potential clinical utility of APPs in the prevention and treatment of infections in infants: (a) APPs may be most helpful in those with reduced levels; (b) during sepsis microbial products signal via pattern recognition receptors causing potentially harmful inflammation that APPs may counteract; and (c) in the era of antibiotic resistance, development of new anti-infective strategies is essential. Evidence supports the potential clinical utility of exogenous APPs to reduce infection-related morbidity in infancy. Further studies should characterize the ontogeny of antimicrobial activity in mucosal and systemic compartments, and examine the efficacy of exogenous-APP formulations to inform translational development of APPs for infant groups.

## Introduction

During early life, the immune system of the newborn (first 28 days of life) and young infant (up to 3 months of age) undergoes remarkable functional change. Historically, the newborn immune system was thought to be an immature version of the adult. However, contemporary evidence suggests that neonatal responses are not simply “immature” but wholly unique, reflecting the distinct immunological needs of fetal versus newborn life (1). Antenatally, the fetus experiences a normally sterile environment until delivery, when the newborn infant is rapidly colonized and challenged with a broad array of microbes (2).

The challenge of immune adaption to this rapid environmental change from immune seclusion to immune challenge may contribute to the propensity of neonates to succumb to overwhelming infection (3). Unique newborn innate and adaptive immunity, reflecting the constraints and needs of the perinatal transition, may also contribute to this susceptibility. Distinct aspects include Th2-polarized responses of monocyte and dendritic cells via pattern recognition receptors (PRRs), T cell hyporesponsiveness to many stimuli (4) and a limited assortment of infant B-cells capable of producing high-affinity antibodies (5). These distinct features of newborn immunity may help prevent overwhelming, and potentially tissue-damaging pro-inflammatory responses and/or potential cross-reactive auto-immune responses to newly encountered microbes. During this immunological transitional period, certain “bridging” mechanisms help provide immune protection for the newborn. This includes “passive immunity” from the transplacental transfer of maternal antibodies to the fetus during pregnancy and postnatal transfer to the newborn primarily through breastfeeding. However, the neonate remains inadequately protected from infection, with over one-third of deaths during the neonatal period directly attributable to perinatal infections, including sepsis, meningitis, pneumonia, and diarrheal disease (6, 7).

**Figure 1:**
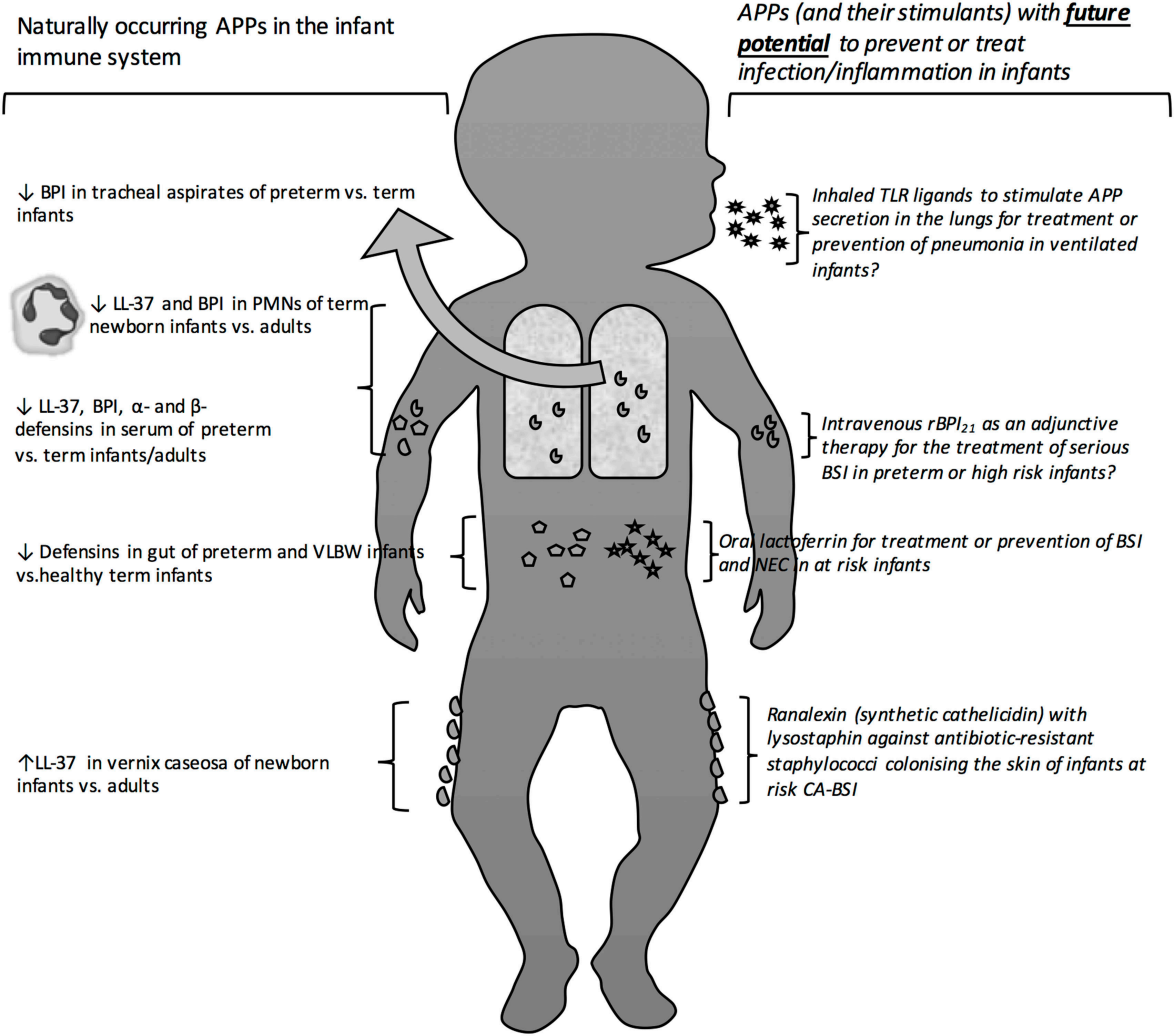
**Antimicrobial proteins and peptides as protective antimicrobial molecules in the newborn bloodstream and at barrier surfaces**. Depicts the site of action of naturally occurring as well as exogenous therapeutic and prophylactic antimicrobial peptides and proteins. Antimicrobial peptide (APP), bactericidal/permeability-Increasing protein (BPI), human cathelicidin (LL-37), polymorphic neutrophils (PMNs), toll-like receptor (TLR), opebacan (rBPI_21_), bloodstream infection (BSI), catheter-associated bloodstream infection (CA-BSI).

In the absence of developed adaptive immunity, infants may particularly depend upon innate immune mechanisms to combat infections. Indeed, primary immune deficiencies, such as MyD88 and IRAK4 defects in the toll-like receptor (TLR) pathways, present in early life, and survival past the neonatal phase is associated with much lower risk of infection (8, 9). Antimicrobial proteins and peptides (APPs) are a key effector arm of innate immunity that function as broad-spectrum anti-infectives against a wide array of Gram-negative and Gram-positive bacteria, mycobacteria, fungi, and enveloped viruses (10–12). In this review, we discuss the capacity of the newborn and infant to express and deploy APPs; how this may affect early life responses to infection; and how exogenous APPs or agents that induce APP expression may have clinical utility in this age group (Figure 1), based on systematically collected data using the methods described in Tables 1 and 2.

**Table 1:** **Literature search strategy**.

Antimicrobial peptide	AND/OR	Infant	AND/OR	Sepsis
Antimicrobial protein		Neonat*		Infection
Lactoferrin		Early life		Pneumonia
Cathelicidin		Newborn		Diarrhea
LL-37		Birth		Necrotizing enterocolitis
BPI				Bacteremia
Cathelin				BSI
HNP-1				Meningitis
HNP-2				Preterm
HNP-3				Prematur*
HBD-1				Low birth weight
HBD-2				Skin
HBD-3				Intestin*
Protegrin				Breast milk
				Amnion*
				Blood
				Lung
				Immun*

*A search for articles was performed using a “systematic review” based method: searching through the Pubmed database using a detailed search strategy with keywords and MeSH terms as listed above. Rapid assessment of the literature to identify the most relevant articles through a rapid screen of titles and abstracts by 1 reviewer. Article selection removed duplicates and the remainder were then screened based on inclusion criteria below*.

Where appropriate * was placed at the end of a group of letters (“trunk” of the word) to retrieve all possible variations.

**Table 2:** **Inclusion criteria for referenced studies**.

Inclusion criteria	
Language	English
Populations	All
Articles that include research on	Antimicrobial protein and peptide (APP) expression and secretion during the first year of life within the blood, mucosal surfaces, and bodily fluids, including preterm, low birth weight, and infected human infants. Clinical trials of APPs as therapeutics that show promise for use in the treatment or prevention of neonatal infections and inflammatory conditions. Where relevant reference animal studies that support clinical studies or hypotheses relating to human infants

*Description of the studies included in the structured review: topics covered and reasons for their inclusion*.

Relative to older infants and adults, newborns (particularly those born preterm) demonstrate lower levels of circulating APPs and reduced cellular release of APPs at sites of infection. This relative deficiency of APPs may contribute to the high risk of invasive infections in early life (13, 14). Factors, such as age, including gestational age at birth, may influence the physiological levels of APPs in infancy. Table 3 depicts APP levels in preterm and term neonates according to anatomical site. Newborns are at increased risk of infection by microbes including fungi (15), Gram-negative bacteria, such as *Escherichia coli (E. coli)* and *Klebsiella pneumoniae (K. pneumoniae)*, and Gram-positive bacteria, such as *Staphylococcus aureus (S. aureus), Streptococcus pneumoniae (S. pneumoniae)*, and *Group B Streptococcus* (GBS) (15).

**Table 3:** **Differential levels of antimicrobial peptides and proteins (APPs) according to age and anatomical site**.

Family/peptide	Site	Sample type	Age groups	APP levels
Cathelicidin: LL37	Blood	Whole blood	Neonates and adults	Lower levels in preterm than term neonates and mothers (enzyme-linked immunosorbent – ELISA) (16). Lower levels in neonatal than in adult neutrophils (flow cytometry), but no difference in plasma levels (ELISA) (17)
	Breast	Breast milk	Mothers	Present in expressed breast milk (EBM) of mothers of term and preterm neonates (reverse-transcriptase PCR (RT-PCR) and ELISA) and in EBM-derived cells (direct immunoprecipitation and western blot) (18, 19)
	Gut	Feces/meconium	Term neonates	Distinct inter-individual variation in feces and meconium (western blot) (20)
	Lungs	Tracheal aspirates	Preterm/term neonates	Detected in bronchoalveolar lavage fluid (BALF) of mechanically ventilated neonates (antigen capture dot-blot assay), concentration did not vary with gestational age (21)
	Skin	Skin biopsies/vernix caseosa	Term neonates and adults	Site-specific expression profile, with expression in human skin biopsies of newborns (immunohistochemistry) (22), higher levels within neonatal foreskin compared to adults (immune staining) (23), and dense expression in vernix of newborns (enhanced chemiluminescence western blot detection system (22) and reverse-phase chromatography-dot blot/western blot analyses) (24)
α-defensins: HNP-1, -2, and -3, and HD-5	Blood	Whole blood	Preterm/term neonates and mothers	Significantly lower HNP-1, -2, and -3 levels in preterm and term neonates compared to mothers (ELISA) (16). Significantly higher HNP-1 and -3 levels in preterm infants delivered to mothers with amniotic infection, compared to normal deliveries (ELISA). Correlation between gestation and HNP levels in preterm infants (25)
	Breast	Breast milk	Mothers	Significantly higher HD5 levels in breast milk from mothers at day 7 than at day 21, and no association between HD5 levels and risk of sepsis (19)
	Gut	Feces/meconium	Term neonates	HNP-1 and -2 in meconium and neonatal feces (ELISA). HNP-3 in meconium (Matrix-assisted laser desorption/ionization-mass spectrometry (MALDI-MS)) (20). HD5 in meconium and feces of neonates (weak cationic exchange chromatography and reversed-phase chromatography/MALDI-MS) (20)
	Skin	Vernix caseosa	Term neonates	The main antimicrobial components in vernix (HPLC, dot blot analysis, mass spectrometry (24), and western analysis (26))
β-defensins: HBD-1 and HBD-2	Blood	Whole blood	Mother–infant pairs	Significantly lower HBD-2 (ELISA) in serum of preterm compared to term infants. Low levels of HBD-2 may be associated with increased risk of late onset sepsis (LOS) (27)
	Breast	Breast milk	Mothers	HBD-1 and HBD-2 levels (ELISA) significantly higher at day 7 than day 21, and displayed antimicrobial activity against neonatal pathogens. No difference between levels fed to infants with and without LOS (19)
	Gut	Feces/meconium	Preterm/term neonates	Similar levels of HBD-2 in preterm and term infants (ELISA), both of which are significantly higher than in children or adults (28). Significant lower levels in feces compared to meconium (ELISA) (29)
	Lungs	Tracheal aspirates/lung tissue	Preterm/term neonates	Present in tracheal aspirates (TA) (antigen capture dot-blot assay) with similar levels in preterm and term infants (21). HBD-2 is the predominant defensin in neonatal lung, and levels (RT-PCR) appear to be developmentally regulated (30)
	Skin	Skin biopsies	Term neonates/adults	HBD-1 is constitutively expressed in human skin (22) and HBD-2 levels comparable between perinatal and adult skin (immunohistochemistry) (23)
BPI	Blood	Whole blood	Preterm/term neonates, and adults	Three- to fourfold lower cellular content of BPI in neonatal compared to adult neutrophils (western blot) (31). Lower plasma levels of BPI in preterm infants compared to mothers (ELISA) (16), and lower ability to release BPI from neutrophils in preterm than term infants and adults (ELISA) (32). Higher levels in infants delivered prematurely due to maternal amniotic infection (ELISA). No association between gestational age and BPI levels (25)
	Lungs	Tracheal aspirates	Preterm/term neonates	Higher levels in term than preterm infants and significant increase in first postnatal week, as detected in acid extracts of neonatal TA polymorphic neutrophils (PMNs) (ELISA) (33)
Lactoferrin	Maternal	Breast milk	Mothers	Most abundant APP present within breast milk of mothers of preterm infants (ELISA) with significantly higher levels at day 7 than day 21 (19)
	Skin	Skin surface/vernix caseosa	Term neonates and adults	Enriched on neonatal skin surface compared to adults (34), and identified in vernix of full-term infants (western analysis) (26)

*Summary of the results of published human studies assessing APP levels in preterm infants, term infants, their mothers, and other adults. Study results are reported in the context of the anatomical site and sample type assessed, the methods used, and the age of the study participants*.

## What are Antimicrobial Peptides and Proteins?

Antimicrobial proteins and peptides are fascinating cationic molecules that are released primarily by neutrophils, monocytes, and macrophages by secretion or during degranulation. APPs are also produced within the skin and at mucosal surfaces by epithelial cells in the respiratory, gastrointestinal, and urinary tract and thus, are present within bodily fluids, including saliva, tears, nasal secretion, gastric juice, sweat, semen, airway surface liquid, and breast milk (35). Clinically important APPs in early life include defensins, cathelicidins, protegrins, bactericidal/permeability-increasing protein (BPI), S100 proteins (e.g., calprotectin), lactoferrin (LF), lysozyme, and RNAses (e.g., 4, 5, and 7) (13).

Defensins are disulfide-rich cationic peptides expressed in plants, insects, fungi, and mammals, including humans (36). Humans express α-defensins (human neutrophil peptides HNP-1, HNP-2, HNP-3, HNP-4, and human defensins HD-5 and -6) and human β-defensins (HBDs including HBD-1, HBD-2, and HBD-3 (28, 37)). Cathelicidins are multifunctional bactericidal peptides with N-terminal fragments bearing a structural similarity to the protease inhibitor cathelin (38), and include human cathelicidin (LL-37), bovine Indolicidin and Ranalexin (39). Protegrins are porcine APPs, structurally similar to cathelins, and have served as templates for development of congeners for therapeutic use in humans (40). BPI is a 456 residue LPS-neutralizing anti-infective protein stored within primary granules of human polymorphic neutrophils (PMNs), and has been developed as a synthetic therapeutic (rBPI_21_) (41, 42). Calprotectin is a predominantly neutrophil-derived metal-chelating protein of the S100 protein family (43), which is gaining recognition as a potential diagnostic marker for necrotizing enterocolitis (NEC). LF is a neutrophil and mammalian-milk derived protein based on one polypeptide chain that contains around 700 amino acids and forms two homologous globular domains (N-and C-lobes) (44, 45).

Antimicrobial proteins and peptides can be constitutively expressed, and/or inducible in response to proinflammatory stimuli. Cathelicidins and HNPs 1–4 are both constitutively expressed and inducible. Lysozyme, LF, HD5-6, and HBD1 are only constitutively expressed, and HBDs 2–4 are only detectable in response to stimuli (46). APPs facilitate effective pathogen clearance by both direct antimicrobial action and immunomodulatory functions (11, 35, 47), inducing angiogenesis, promoting wound healing (10), inhibiting LPS-induced proinflammatory responses (10, 48), modulating adaptive cellular immune responses (13, 49), mediating immune cell ontogeny in the lung and gut, and acting as chemoattractants for other immune cells. Chemokines and cytokines regulate the release of APPs but can also display direct antimicrobial activity themselves: indeed, up to two-thirds of human chemokines have been shown to have some direct antibacterial action (46).

Antimicrobial proteins and peptides target invading bacteria via initial electrostatic contact at the anionic bacterial surface. The specific mode of action differs between APP families but permeabilization of target cytoplasmic membranes is a common crucial step in APP-mediated antimicrobial activity and cytotoxicity (47). The concept of extracellular entrapment of bacteria, and the contribution of APPs to this process, has advanced in recent years, both in relation to antibacterial activity at epithelial surfaces and within the bloodstream. Yost et al. describe neutrophil extracellular traps (NETs), which are lattices of extracellular DNA, chromatin, and APPs that mediate extracellular killing of bacteria (50). A similar process occurs at the intestinal mucosal surface whereby defensins form nanonets to trap bacteria and combat invasion across the intestinal barrier into deeper tissues (51).

APPs have potential as stand alone therapeutics or as adjunctive agents, to reduce either length of antibiotic treatment and/or inflammation induced by killed microbes/microbial products (52). Important APPs that have undergone clinical trials include rBPI_21_; Pexiganan, an analog of Magainin (MSI-78) (53); Iseganan (IB-367), a protegrin mimetic; Omiganan pentahydrochloride (CLS001), an Indolicidin analog; Brilacidin, a defensin mimetic; LTX-109, a short lactoferricin-based peptide (54), and Talactoferrin, an analog of LF (55, 56). The terms analog and mimetic are used, respectively, to describe synthetic compounds with closely similar molecular structure versus those with a closely similar functional capacity to that of an endogenous APP (see Table 4).

**Table 4:** **Important synthetic antimicrobial proteins and peptides according to the endogenous compounds from which they are derived**.

Origin	Endogenous compounds	Exogenous synthetic compounds
Bovine	Indolicidin Bovine Lactoferrin (BLF)^b^	Omiganan pentahydrochloride (CLS001) LTX-109
Porcine	Protegrin (β-defensin 2 (pBD-2)	Iseganan (IB-367) pBD-2^a^
Amphibian	Ranalexin Magainin	Polymixin Pexiganan (MSI-78)
Human	Human defensins Cathelicidin Bactericidal/permeability-increasing protein (BPI) Lactoferrin (LF)	Brilacidin N/A rBPI_21_ Talactoferrin and LTX-109

*^a^Denotes promising compounds currently in pre-clinical experimental stages, all other synthetic forms are in clinical trial stages*.

*^b^Denotes compounds in the endogenous form that have undergone human clinical trial*.

## APPs and the Skin: At the Frontline of Immune Defenses

Many APPs exert their main effects at the frontlines of the body’s immune defenses – the skin and mucosal surfaces. The skin acts as both a physical barrier and a chemical barrier to potential pathogenic organisms hosting an array of APPs including LL-37, LF, a- and b-defensins (see Table 3). HBD-2 is particularly effective against both Gram-negative and Gram-positive bacteria, such as *E. coli* and *S. aureus*, respectively (40). Amphibian skin has proven to be a promising source of new APPs (57), which can be chemically synthesized for human use. For example, Pexiganan (an analog of magainin – isolated from the skin of the African clawed frog) shows promise for use in the treatment of localized skin infections in humans (53). *Staphylococcus epidermidis* (*S. epidermidis*) is commonly found on the skin, and is responsible for clinically significant infection in preterm and low birth weight (LBW) infants (58). LL-37 significantly inhibits growth of *S. epidermidis* isolated from the skin of newborn infants (59), and the reduced levels of LL-37 in preterms (6) may contribute to their susceptibility to *S. epidermidis* infection. In fact, in newborn infants, the lesions of a commonly encountered harmless rash only seen in the neonatal period, termed as “erythema toxicum,” are densely filled with LL-37 expressing neutrophils and eosinophils. While the exact trigger for the erythema toxicum rash remains unclear, it appears that activation of innate immune cells to express APPs occurs and, thus, colonization of the skin with microbial flora may initiate this process (22).

## APPs in the Lungs and During Pneumonia

Airborne organisms can gain entry to the human host through the airways, but the lung tissue is well protected from invasion by epithelial lining fluid that is rich in APPs (60); including LL-37, defensins, and lysozyme (32). Resident mucosal immune cells (e.g. alveolar macrophages), epithelial cells, and systemic immune cells (recruited to the lung epithelium at times of microbial challenge) all contribute to the secretion of APPs into epithelial lining fluid (33). During an episode of pneumonia, increased levels of APPs are detectable in bronchoalveolar lavage (BAL) fluid (21). It is clear that HBD-2 is the predominant defensin in neonatal lung, and whether defensin levels are lower in preterm or term infants is yet to be established (30). However, there appear to be reduced levels of BPI in the lungs of preterm infants compared to term infants, which may contribute to the higher risk of pneumonia in this age group; with lower lung APPs, preterm infants may be unable to clear pathogenic organisms effectively (30, 33, 61).

## APPs in the Intestine in Health and Disease

The human intestine harbors a broad array of micro-organisms (the intestinal microbiome), which are increasingly understood to interact dynamically with the host immune system potentially leading to long-term effects on health. APPs are believed to significantly alter environmental microbiota and influence expression of pattern-recognition receptors at the intestinal epithelial surface (62). Indeed, mouse models have helped describe the homeostatic role of α-defensins in regulating the makeup of the commensal microbiota in the neonatal intestine (63). However, while hosting beneficial bacteria, the intestinal mucosa must also protect itself from dangerous invasive organisms: Paneth cells contribute to this protection by secreting defensins and other APPs into the intestinal fluid. The various mechanisms by which gut defensins in particular are able to protect the intestinal mucosa from microbial invasion continue to be elucidated. Recent work published in *Science* describes eloquently how HD-6 released from Paneth cells undergoes a complex self-assembly into nanonets and fibrils at the ostia of crypts, allowing highly effective entrapment of bacteria and preventing damage to stem cells at the base of crypts (51).

There is a paucity of literature describing APP function in the healthy human newborn and infant gut, but some inferences can be made from studies of animals, and of human fetal tissue. Perhaps contrary to what we might expect, a recent study indicates that specific APP levels are increased in the mouse intestine during the neonatal period. The intestinal intraepithelial cell (IEC) mRNA expression levels of the mouse cathelicidin-related antimicrobial peptide (mCRAMP), the murine intestinal homolog of human LL-37, is highly expressed in healthy term neonatal epithelium and becomes less abundant during the postnatal period as IEC proliferation and differentiation occurs (64). Indeed, mCRAMP expression has previously also been shown to be increased in embryonic and neonatal mouse skin, when compared with adult skin (23). Further research is required to explore whether this specific developmental phenomenon exists in the skin and intestinal mucosae of human infants.

Conversely, studies of human fetal intestinal tissue support the premise that APP levels are relatively diminished in early life: reduced mRNA expression levels of HD-5 and HD-6 have been reported within terminal ileal tissue at 24-week gestation compared to full-term infants and adults (65). Indeed, data suggest that low levels of defensins in preterm infants are associated with increased incidence of intestinal pathology, in particular the devastating illness, Necrotizing enterocollitis (NEC) (66). NEC etiology is incompletely understood but an interplay exists between host factors [prematurity, very low birth weight (VLBW)], the intestinal microbiota and enteral feeds. APPs potentially contribute, as animal models have shown that depletion of Paneth cells of α-defensins followed by enteric infection results in a clinical picture akin to human neonatal NEC (67).

Ileal tissue from infants with NEC show elevated defensin levels compared to age-matched controls, likely indicating that at some stage in the pathogenesis of the disease, Paneth cells are induced to increase production of defensins (65). Higher HBD-2 concentrations appear to have a protective effect once NEC pathology is established, in that they have been associated with more moderate courses of the disease. Indeed, in severe NEC, low HBD-2 expression is accompanied by low TLR4/MD2 expression, suggesting an inadequate response to luminal bacteria, possibly predisposing to the development of NEC (29). Calprotectin levels have been extensively investigated in neonatal stool samples, as a potential screening marker for the detection of NEC. A recent systematic review of the literature confirmed fecal calprotectin levels are elevated in NEC, but whether this is robust enough to act as a diagnostic test early in the disease, and what relevance the levels have to disease progression and severity remains unclear (43).

An important mode by which the infant intestinal mucosal surface is furnished with APPs, is through ingestion of maternal breast milk. APPs may contribute to the ability of breast milk to protect the newborn from inflammatory and infectious diseases. Several APPs have been identified in breast milk, including LF (68), lysozyme (69), LL-37 (18), α-, and β-defensins (19, 70). Importantly, LF is abundant at concentrations sufficient to inhibit bacterial growth (19, 70). Given its multi-functional immuno-modulatory, anti-inflammatory, and antimicrobial properties, LF supplementation in VLBW has been increasingly studied for the prophylactic treatment of bloodstream infection (BSI) and NEC (71). Evidence supports the notion that combinations of components working in synergy contribute to the antimicrobial activity of breast milk, as exemplified by the concomitant action of LF with bovine RNase 5 (angiogenin-1), RNase 4, and angiogenin-2 (72). A strategy of mimicking the synergistic nature of breast milk derived APPs and associated molecules has potential for future therapeutics.

## APPs in the Blood and Bloodstream Infection

APPs consistently circulate in the bloodstream, they are transported freely within the plasma, and provide an ongoing low-level non-specific immune defense against potential invasive pathogens. Cellular expression and secretion of some APPs, including defensins (73), LL-37 (74), and BPI can be mediated by TLRs (6). In infants with BSI with bacterial etiology, plasma BPI concentrations are higher than those in healthy infants, which indicates that BPI transcription and/or cellular secretion is upregulated during infection (41, 75). Additionally, healthy uninfected neonates born to mothers who have suffered from an amniotic infection demonstrate higher levels of LF, BPI, HNP-1, HNP-2, and HNP-3 in the cord plasma (25). Maternal plasma LL-37 levels appear to be the most important predictor of infant plasma LL-37 levels, and although the source of these APPs in cord blood is not known, it is possible that these higher levels may not be a reflection of the functional status of the infant’s own immune system, but an example of maternally derived transplacentally transferred immune protection (76).

However, generally, intracellular levels of APPs are lower in neonates than in later life: LL-37 and BPI levels are reduced in neonatal whole blood and neutrophils when compared with adults (17, 31, 32, 77), and BPI deficiency of neutrophils in neonates is associated with reduced bacterial-killing capacity (41). It is yet to be established whether an infant’s intrinsic intracellular or plasma levels of APPs influence an individual’s risk of developing a BSI, or indeed the clinical outcome following BSI. Measuring serum, plasma or even resting-state intracellular levels of APPs have obvious limitations in understanding the importance of differences between neonates and adults. Indeed, more relevant perhaps is identification of functional impairments of the innate immune response in neonates, such as defective NET formation resulting in impaired bacterial killing *in vitro* (78). Further characterization of the functional capacities of peripheral blood neutrophils in term and preterm infants will undoubtedly yield insights into understanding neonatal BSI and developing strategies for its prevention and cure.

## The Premature Infant: A Special Case

Premature birth significantly increases susceptibility to serious infections, including BSI, meningitis, and pneumonia (79, 80). APP levels are generally lower in preterm than in full-term infants, including within the bloodstream (16) (both in the circulating plasma and intracellularly within immune cells), at epithelial surfaces and within bodily fluids and feces (6, 29, 30) (Table 3). This relative deficiency in APPs may contribute to the preterm infant’s increased risk for invasive bacterial infection (13). Importantly, higher levels of APPs (including HBD-1, HBD-2, and LL-37) are seen in the blood and body fluids of those with acute infections, such as BSI (81) and respiratory infection (21). Increased levels of APPs in cord blood of infants born to mothers with a history of BSI or chorioamnionitis (25) is important in the context of premature infants, as preterm delivery is often triggered by amniotic infection that may, therefore, act as a significant confounder when assessing for effect of gestational age on APP levels. Studies not taking a history of chorioamnionitis into account should, therefore, be interpreted with caution (21, 28).

## Clinical Application of APPs: Promising Evidence from Clinical Trials in Adults?

While the number of APPs undergoing pre-clinical development has been increasing, the majority of clinical trials have focused on topical formulations with few trials in the pediatric population (Table 5). Several lead compounds, including Pexiganan, Iseganan, and Omiganan have failed to achieve late stage development due to their failure to meet primary trial endpoints, or disappointingly insurmountable regulatory hurdles (56). Currently, Dipexium Pharmaceutical’s Locilex (Pexiganan cream 0.8%) is the only APP undergoing a phase III clinical trial, for the treatment of mild wound infections (NCT01594762). Cellceutix Corporation recently completed phase II trials of Brilacidin in acute bacterial skin infections (NCT02052388) and have begun preclinical studies in otitis media and ocular infections. Cutanea Life Sciences has identified new indications (including skin infections) for Omiganan (CLS001), which was previously not approved for urinary tract infections (NCT02456480). Lytix Biopharma has completed phase II trials of LTX-109 in impetigo (a problematic condition primarily affecting young children). Several pharmaceutical companies are developing APPs for systemic administration, such as Agennix AG who are pursuing the development of oral Talactoferrin in severe sepsis (NCT00630656). Results from their phase II randomized controlled trial (RCT) showed a significant reduction in all-cause mortality at 28 days and 6 months in the treatment group (82) yet, the recent follow on phase II/III RCT (OASIS trial) was terminated prematurely over concerns of safety and efficacy (55). Interestingly the APP that has undergone most advanced clinical testing using the intravenous (IV) route is rBPI_21_, which was assessed for its efficacy in meningococcemia in children (42). Other AMPs, such as LF 1-11 (hLF1-11), are undergoing safety and tolerability testing for delivery via the IV route in healthy volunteers (83).

**Table 5:** **Antimicrobial peptides and proteins evaluated in clinical trials for the treatment of infections in children**.

Peptide	Clinical application	Treatment arms (*n*)^a^	Phase^b^	Status	Company	Outcome	Reference/Reg no.^c^
Opebacan (rBPI_21_): recombinant 21-kDa modified fragment of human bactericidal/permeability-increasing protein (BPI)	Severe meningococcal sepsis	rBPI_21_ (190) Placebo (203)	III	Complete	Xoma	The trial was underpowered to detect significant differences in mortality. However, patients receiving rBPI_21_ had a trend toward improved outcome in all primary outcome variables, and the study authors concluded that rBPI_21_ is beneficial in decreasing complications of meningococcal disease	Levin (42)
Bovine lactoferrin (BLF): 80 kDa naturally occurring multifunctional glycoprotein of the transferrin family	Late-onset sepsis	BLF (153) BLF plus LGG (151)	NS	Complete	Saint Anna Foundation and Dicofarm	Compared with placebo, BLF supplementation alone or in combination with LGG (*Lactobacillus rhamnosus* GG) reduced the incidence of a first episode of late-onset sepsis in VLBW neonates (84). Prophylactic oral administration of BLF also reduces the incidence of invasive fungal infection in preterm VLBW neonates	ISRCTN53107700; Manzoni (84, 85, 86)
	Invasive fungal infections	Placebo (168)					
	Necrotizing enterocolitis	BLF (247) BLF plus LGG (238) Placebo (258)	NS	Complete		Compared with placebo, BLF supplementation alone or in combination with LGG reduced the incidence of ≥stage 2 NEC and of death-and/or ≥stage 2 NEC in VLBW neonates (86)	
	Late-onset sepsis Necrotizing enterocolitis	BLF (22) Placebo (25)	NS	Complete	Ankara University	Fewer sepsis episodes were observed in LF-treated infants with none developing NEC, without statistical significance (87)	NCT01287507; Akin 2014 (87)
	Late-onset sepsis	BLF (95) Placebo (95)	II	Complete	Universidad Peruana Cayetano Heredia	Overall sepsis occurred less frequently in the LF group than in the control group. Although the primary outcome did not reach statistical significance (88)	NCT01264536 (88)
		BLF Placebo	III	Ongoing			NCT01525316
	Healthcare-associated infections Necrotizing enterocolitis	BLF Placebo	NS	Complete	Research Center of Sainte Justine, Canada	Results awaited	ISRCTN66482337
	Late-onset sepsis	BLF Placebo	III	Ongoing	National Health and Medical Research Council, Australia	Results awaited	ACTRN12611000 247976
	Late-onset sepsis	BLF Placebo	III	Ongoing	The National Institute for Health Research, UK	Results awaited	ISRCTN88261002

*^a^BLF, bovine lactoferrin; LGG, probiotic *Lactobacillus rhamnosus* GG; GOS; galacto-oligosaccharides. Sample size (*n*) is absent for trials either in progress or completed but unpublished*.

^b^NS, “not stated.”

*^c^Reference or registration numbers are obtained from http://clinicaltrials.gov, http://www.isrctn.com, and http://www.anzctr.org.au*.

## Clinical Applications of APPs in Infants

Evidence supports the use of recombinant congeners of APPs to improve circulating levels and potentially reduce the incidence of, and/or improve outcomes from bacterial infection in infants. APPs have been used to prevent infections and aberrant inflammation in high-risk infants, such as premature and LBW infants. A large, multicentre, double-blind, RCT comparing LF supplementation alone or in combination with probiotics demonstrated a significant reduction in late onset sepsis (LOS) in VLBW infants in both treatment groups as compared to placebo controls (84). Secondary analysis of data from the same RCT showed significant reductions in incidence rates of invasive fungal infection in both treatment groups as compared to placebo controls (85). A third LF study demonstrated that the same treatment interventions significantly diminished incidence of NEC in VLBW infants (86). These findings were reiterated in a smaller RCT which found that LF-treated infants experienced fewer primary but also secondary episodes of sepsis as compared to placebo (87). A recent Peruvian study also reported that infants receiving LF were less likely to develop sepsis than placebo controls (88). Taken together, these findings highlight the feasibility of supplemental LF, either alone or in combination with probiotics, as a promising approach to protect VBLW infants from neonatal infections.

Invasive meningococcal disease is a rare but devastating disease, associated with high morbidity and mortality in the young. Promising preclinical data supported the antibacterial and anti-endotoxin properties rBPI_21_, while phase I/II trials demonstrated the safety of rBPI_21_ in adults and suggested beneficial effect on inflammatory biomarkers in children with severe meningococcal sepsis, thus prompting a phase III RCT for this indication (89). The study, whose youngest participant was ~2 weeks old, suggested that rBPI_21_ conferred benefit with respect to mortality and morbidity. By intention to treat analysis, mortality was lower in the rBPI group, though not significantly. A sub-group analysis of those who survived to complete the first infusion of rBPI or placebo demonstrated nearly a 50% reduction in mortality in the rBPI_21_ group. Although the study was underpowered to detect significant changes in mortality by intention to treat analysis, rBPI_21_-treated study participants had a substantial reduction in severe limb amputations, shorter ICU stay, and better return to baseline function. These results suggest the potential utility of rBPI_21_ in reducing meningococcal-associated complications and, if approved such that it could be given even sooner in the sepsis cascade, would likely confer even greater benefit. Notably, when administered to total body irradiated mice, rBPI_21_ demonstrated benefit in conjunction with conventional fluoroquinolone antibiotic, including more rapid recovery of the hematopoietic compartment and improved survival suggesting that it may be a useful adjunct in those deficient in BPI due to chemoradiotherapy (90). These results raise the possibility to extend the beneficial effects of rBPI_21_ to other populations that are relatively deficient in functional BPI activity, including the preterm infant group.

## Future Potential of APPs in Diseases of Infancy

Circulating and intracellular levels of APPs are relatively low in early life, especially in those born preterm or with LBW, potentially contributing to susceptibility to infection. There are several rationales for the potential clinical utility of APPs in the prevention and treatment of infections in infants: (a) APPs may be most helpful in those with reduced levels; (b) during sepsis microbial products signal via PRRs causing potentially harmful inflammation which APPs may counteract; and (c) in the era of antibiotic resistance, development of new anti-infective strategies is essential.

Clinical trials of oral LF and IV rBPI_21_ have suggested significant clinical benefit lending support to the hypothesis that APPs, either induced endogenously or as exogenously administered congeners, may help prevent and treat infections in highly susceptible infants in early life: particularly premature or VLBW infants. Future strategies should identify and develop APPs with potential for prevention and treatment of the most devastating diseases: BSI, pneumonia, CNS infection, diarrheal disease, and NEC. There are a number of strategies that have as yet made little progress in clinical trials: such as inhaled TLR ligands that can stimulate production of APPs at the lung surface. The company *Pulmotech* have developed “PUL-042”; a novel combination of two synthetic TLR agonists (Pam2 and ODN) (91) that will begin Phase 1b/2a clinical studies this year in immunosuppressed adults at high risk of developing pneumonia. PUL-042 or similar compounds could be considered for use to reduce pneumonia in at risk infants, such as ventilated premature or VLBW infants.

## APPs in the ERA of Antibiotic Resistance

In the era of antibiotic resistance individual APPs, combinations of APPs, or agents that induce their expression (e.g. TLR agonists), may serve as novel alternatives to antibiotics. It has been proposed that bacterial resistance to APPs is much less likely to evolve than to conventional antibiotics, owing to their broad, non-specific antibacterial mechanism of action (92). The *in vivo* response to infection involves the action of multiple endogenous APPs and, thus, a combination therapy of multiple synthetic APPs may be a better therapeutic option than an individual agent.

As long-term survival rates of preterm and VLBW infants in neonatal intensive care units (NICUs) increase, so does morbidity associated with catheter-associated blood-stream infections (CA-BSIs). Neonates are at particular risk of exposure to antibiotic resistant bacterial BSIs: specifically, from, *methicillin-resistant Staphylococcus aureus* (MRSA), vancomycin-resistant enterococci (VRE), and extended spectrum beta-lactamase producing Gram-negative bacteria (ESBL) (93). New strategies are needed to eradicate antibiotic-resistant bacterial strains, including those colonizing or infecting the skin and mucosal surfaces, before the organisms gain entry to the bloodstream. Experimental data are emerging on the potential of APPs as single or synergistic agents to existing therapies in this regard.

In the case of staphylococcal-resistant organisms, a recent study using a skin explant model to assess the efficacy of Ranalexin with an endopeptidase (Lysostaphin) found that the combination was able to rapidly and specifically kill resistant staphylococcal species without adversely affecting normal skin microflora (94). Additionally, a group from Singapore have designed four hybrid peptides (based on Indolicidin and Ranalexin), which display strong antibacterial activity against MRSA *in vitro* (95), and another *in vitro* study identified Indolicidin (and a number of other APPs) alone and in combination with antibiotics, as potential candidates for future therapeutics against MRSA biofilms (96). The underlying mechanisms explaining these synergistic effects against MRSA remain to be completely elucidated. An *in vitro* and *in vivo* study of Nafcillin (an anti-staphylococcal β-lactam) identified that it enhances killing of MRSA by increasing the binding of LL-37 to the MRSA membrane.

Infection with penicillin-resistant strains of *S. pneumonia* can be a serious therapeutic challenge in the young infant. Recently, a Malaysian research group designed a novel hybrid peptide “DM3” that has shown synergistic therapeutic efficacy in combination with penicillin in a mouse model of systemic infection with a strain of penicillin-resistant *S. pneumoniae* (97). Design and testing of APPs to ensure maximal efficacy while limiting toxicity is of paramount importance for the vulnerable infant age group.

## A Broad-Based Approach to Future Research

Newborn infants and in particular those born prematurely are highly susceptible to invasive and often overwhelming sepsis. Data from the World Health Organization suggest that worldwide every year 1.1 million neonates die from infection (3). Evidence is growing for the potential of APPs to be useful in the reduction of morbidity and mortality from infection in infancy in both resource-rich and resource-poor countries. Further research is needed, including *in vitro* and *in vivo* studies characterizing the ontogeny of global cellular and soluble antimicrobial and anti-infective (e.g. endotoxin-neutralizing) activity within systemic compartments and at epithelial surfaces. These basal surveys will then need to be systematically compared in relation to induced and exogenous-APP supplemented fluids to inform translational development of APPs in high-risk populations, including newborn and infant groups.

## Author Contributions

AB, OL, and BK contributed to the conception of the review article. AB, JK, and VW undertook the literature review and identified key papers for inclusion. AB and JK drafted the initial article, and OL, VW, and BK contributed to all revisions and subsequent drafts. All authors have given final approval for the version submitted for publication.

## Conflict of Interest Statement

The authors declare that the research was conducted in the absence of any commercial or financial relationships that could be construed as a potential conflict of interest.
